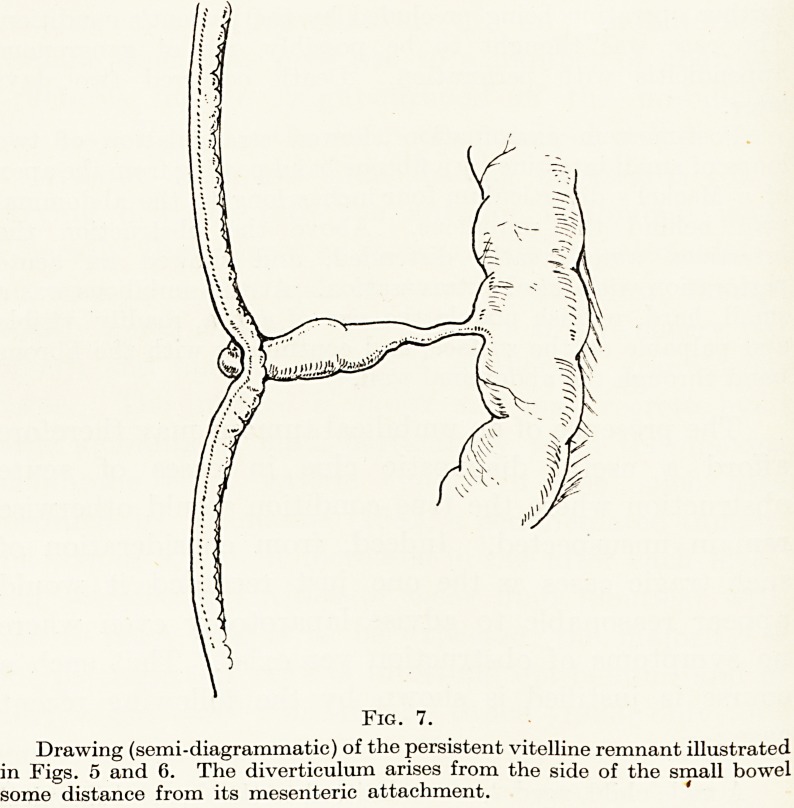# Some Intestinal Malformations and Their Clinical Significance
*Communicated to the Bristol Medico - Chirurgical Society, 8th April, 1931.


**Published:** 1931

**Authors:** Arthur L. Taylor

**Affiliations:** Pathologist, Bristol General Hospital; Demonstrator of Pathology, University of Bristol


					PLATE II
JL '*
E-M-W- ?
Fig. 1.
Islets of aberrant gastric mucosa
in the upper end of the oesophagus,
closely resembling areas of shallow
ulceration. A fairly common post-
mortem finding and of doubtful
clinical importance.
Fig. 4.
Nodule of accessory pancreatic
tissue invaginating the tip of &
Meckel's diverticulum to form the
apex of an intussusception. The
diverticulum is turned completely
inside out, and its mucous membrane
is turgid and deeply congested from
strangulation of its vessels.
Figs. 5 and 6.
a. Patent vitelline duct of varying thickness, continuous with a
Meckel's diverticulum and associated with a small umbilical fibrous nodule,
through which a small faecal fistula had discharged since birth.
b. The same, laid open longitudinally, showing a complete lining of
mucous membrane which is somewhat atrophic in its distal part.
The Bristol
Medico-Chirurgical Journal
" Scire est nescire, nisi id me
Scire alius sciret
SUMMER, 1931.
SOME REMINISCENCES AND A FORECAST.*
BY
Sir Ewen J. Maclean, M.D., D.Sc. (Hon.), F.R.C.P.,
F.A.C.S. (Hon.), F.R.S.E.,
Professor of Obstetrics and Gynaecology, Welsh National School
of Medicine;
Vice-President, British Medical Association, etc.
Bristol has always seemed to me to typify in her
professional classes, her business men, and in her social
endeavour that combine of solid worth, steady,
unfaltering grip, whether in good times or bad, and
unheralded point to point attainment, which is summed
up in that great word England. We may not wholly
agree with Thomas Carlyle that " The English are a
dumb people. They can do great acts but not describe
them " ; but there is something wholly admirable and,
indeed, comforting to the more vocal and demonstrative
Celtic fringe in the wise, almost parental tolerance of
the predominant partner.
* An address delivered to the Bristol Medico-Chirurgical Society on
Wednesday, 11th February, 1931.
Vol. XLVIII. No. 180.
84 Sir Ewen J. Maclean
My personal knowledge of Bristol began in the last
century?I do not mean a century ago, but in 1890?-
when I was advised to gain some knowledge of general
practice by accepting an assistantship in the Lawrence
Hill district, and I recall that my then very youthful
appearance created some difficulty in my being received
as a substitute for my chief by the actively parturient
ladies of the East End. In relation to this experience,
not altogether rare amongst young practitioners, a
senior chief of mine later said to me : " Your physical
make-up, Maclean, is like mine. For years I felt
handicapped by the impression that I looked too
young until one day I overheard someone say that I
was too old. Believe me, young man, subjectively,
there is no intermediate stage ! " There was, however,
some consolatory glamour in the fact that not
infrequently the great W. G. Grace and I met to
minister to the sick in the same street?though not
in the same house, and not, of course, for the same
firm.
Then I was House Surgeon for over two years at
the Bristol Royal Hospital for Sick Children and
Women on St. Michael's Hill, and was a member of
this Society, and I trust the records show that I duly
paid my subscription. Much to my profit, I was and
still am a subscribing member to the Journal, which
owes so much in its development to the late L. M.
Griffiths, the memory of whose qualities and zeal are,
I know, still held high among you. The Society
assembled in the Old Library, the premises of which
were nothing like so palatial or impressive as the
beautiful building of to-day; but I would venture to
claim that the functioning of the Society, in all
Some Reminiscences and a Forecast 85
essentials, could not be bettered. It was there that
I, with other recently qualified men, met and was
privileged to know the leading members of the
profession, and I shall always gratefully remember
the kindness and encouragement extended to us as
juniors. If I may say so, as succeeding years have
conferred the inevitable senior status upon me I
have endeavoured to follow their example?surely
one of the prime objects of our societies. To mention
only a few names, I especially recall Edward Long Fox,
Shingleton Smith, Markham Skerritt, Greig Smith,
and last but not least, and still happily with us,
Richardson Cross.
I came to Bristol from my Alma Mater, Edinburgh
University, where in matters surgical antisepsis was
dominant, and had not, as yet, given due place to
asepsis. The smell and hiss of the carbolic spray
assailed ears and nostrils in all the hospitals of the
land, and .those in Bristol afforded no exception.
Buried sutures, including those of rather crudely
prepared catgut, were coming more into vogue, and
sometimes over-reaching the absorptive capacity of
the tissues. Chicken-bone drainage tubes in osteotomy
cases failed to disappear in the time and manner it
was intended they should, and contributed to some
unsatisfactory results. At the Children's Hospital I
made myself responsible for the collection and
preparation of the chicken bones of appropriate size
and shape, and the response of my social friends
became so cordial and active in the matter of collection
as to create some embarrassment and even to affect
appreciably the corresponding poultry market?as
was jocularly suggested.
86 Sir Ewen J. Maclean
It is interesting to recall the then phase of the
treatment of tuberculosis by Koch's tuberculin, which,
discarding the precautions and reservations which
accompanied the pronouncement by that great man,
spread like putting a match to the heather, with, in
many instances, devastating results. I obtained leave
to witness the methods then employed in London. A
very distinguished friend of mine was amongst the
victims who succumbed to the unguarded enthusiasm
of the movement before a more matured experience
determined the limits of safety and utility.
I made many friends amongst the resident staffs
of the Bristol Royal Infirmary and the Bristol General
Hospital, and it was not difficult to discern the friendly
rivalry between the two institutions, which was rooted
in the remoter days when the feuds between
Dolphinites and Anchorites were affairs of even more
than wind and limb. No member of this Society, I
imagine, is unacquainted with Augustin Prichard's
brochure on " The Early History of the Bristol
Medical School," in which it is related that the Bristol
Royal Infirmary being the first of the two hospitals to
obtain royal recognition, a certain Mr. Sanders, whose
sympathies with one of them were not disguised, gave
expression to the historic adage that " The patients
who want a Sovereign remedy will now go to the Royal
Infirmary ; but those who want a Radical cure will
go to the Hospital ! " To what extent, if any, that
injunction was at that time acted upon or has since
been observed, it is not for a privileged guest to inquire
<or even to surmise.
It would appear that in those days the partisanship
of the Bristol layfolk in matters medical was to some
Some Reminiscences and a Forecast 87
extent political, and Augustin Prichard submits?to
quote again from his pamphlet ? an interesting
observation on their attitude toward movements other
than commercial. He says : " There seem to be some
slight signs abroad that the people of Bristol are
beginning to shake off some of that lethargy in the
matter of science, literature, and the arts which has
been so conspicuous for the last half century, and to
feel some care for things beside trade, commerce, and
politics ; and in producing this most desirable change
the University College and Medical School must be
allowed to have had a considerable share, and will,
I hope, receive a corresponding reward in their
future progress." As we view to-day the munificent
benefactions of your merchant princes, and in particular
those of the Wills family, we may truly claim that
Mr. Prichard's faith has been lost in sight and his-
hope in abundant fruition.
Evolution, whether social or scientific, will not
be denied its advances, and whilst for long periods it
may be unseen to all but a few discerning souls, it
has a way of developing a phase of full and dominant
expression. In no sphere of thought and work has
this been more exemplified than, during the past half
century, in the art and science of medicine.
The measures of medicine have been filled to'
overflowing by the contributions of our own workers
and those of the sister arts and sciences. New facts,
and new settings of truths long known have been
submitted for transmuting into agencies for the
prevention, alleviation and cure of disease. The mass
of knowledge has grown so great that the curriculum
for our students, though much extended in time, still
88 Sir Ewen J. Maclean
remains congested. In practice this has become
expressed in the divisions and sub-divisions of
specialism, so that it is sometimes said that the
specialist gets to know more and more about less and
less and the general practitioner less and less about
more and more. Accumulated knowledge, no doubt,
supplies the vis a tergo in the creation of new departments
of practice, but there is also a powerful vis a froute
in the public demand for specialist treatment. In
the existence of a rising market for such services one
of the dangers is the ready-made or even the
reach-me-down specialist ? the outcome of something
like mass production out of a hasty curriculum in
which a student may as early as his second year
declare his suit as a prospective specialist and thereupon
cause his subsequent training?largely theoretical?-
to be correspondingly focused, much to the detriment
of that fundamental principle that all sound and
justifiable specialism must be based on a thorough
general training.
The General Medical Council has done much to
prevent any marked degree of such untoward
developments in this country, but I have personally
observed them in important medical centres in other
lands, where they are so rife as to cause grave concern
to some of their directors. We have, in our own
country, a more rational reaction to the call for the
harnessing of new agencies for the diagnosis and
treatment of disease, in the shape of team work in
general practice, in consulting practice and in
institutional service. In its optimum form this is
conducted by well-trained and experienced practitioners
and researchers.
Some Reminiscences and a Forecast 89
The articles and correspondence columns of our
medical journals reveal divergence of opinion as to
whether the clinically-minded man and the laboratory-
minded man can work in effective unison, or whether,
in the alternative, we must await the evolution of a
new order of men so gifted and ordained as to be
capable of putting on more than a little both ways.
Some of us know amongst our research colleagues
men for whose abilities and personalities we have the
highest regard who would be as much at sea in a
hospital ward as we should be amid the tangle and
equipment of their laboratories.
Of our liaison colleague, the pathologist, we know
much. He is, so to speak, one of us, and is quite often
indispensable in matters of clinical diagnosis. We
are familiar, too, with his demeanour of benevolent
reproach when we attend, shall we say, an occasional
sectio cadaveris to have our shortcomings pointed out
to us. But what about our more recent colleague, the
bio-chemist, to whose hands it would seem is entrusted
the great objective of revealing the utterly complex
factors which constitute, and the conditions which
vary resistance to disease and the various infections ?
As clinicians we do not know so much of him, but now
that he has been summoned from the empyrean to
deal with our earth-bound spirits, we confidently hope
both to know and to appreciate him, and to introduce
him to our patients, if such be his desire. Can our
bacteriologists get much farther without some such
revelation as that referred to, or will they, without
waiting upon the bio-chemist, break through into the
unknown themselves and get on with the business ?
If so, let them be assured the clinical hosts will
90 Sir Ewen J. Maclean
follow them hot-foot, and help to at least widen the
breach.
Meanwhile, taking the case of septic infection, for
example, the clinicians are " milling around" in a
space from which there seems, for the time being, to
be no escape. And the bacteriologists, to keep us
moderately quiet, in the absence of further discoveries,
keep on splitting up the streptococci into an ever-
increasing number of groups with about as much
affinity as exists, for example, between North and
South Wales, or between Lancashire and Yorkshire,
or even Devon and Cornwall. In our patience we
will do our best to possess our souls.
If so much may be submitted regarding the trend
of scientific evolution as it affects us, what may be
said of the evolution of the legislative frame-work
set up by the State during the past twenty years, and
in which the profession is required to discharge its
duties to the public ?
I suppose that, with one possible exception, there
is no other country in the world in which, rightly
understood, so much has been done in this direction,
and, if I read aright the portents of recent legislation,,
in which so much more is being and will be done.
Presumably it is the relative compactness of the
British Isles and the density of their population?
two factors which are at once our strength and, in
some respects, our weakness?which largely account
for this priority of administrative evolution.
The re-alignment of the conditions of general
practice brought about by the Health Insurance Acts
?a vivid memory to some of us?amounted to
something of the nature of a revolution ; bloodless,.
Some Reminiscences and a Forecast 91
it is true, but sufficiently disturbing at that. A
majority opinion will acclaim those Acts, after eighteen
years' experience of their operation, as on the whole
successful so far as they go, but equally it will be
conceded that they cannot be regarded as other than
a veritable torso in relation to a full-bodied National
Health Scheme. Fuller developments are, of course,
inevitable, and some of them of comparatively minor
importance are already available in the form of
additional benefits granted by certain of the Approved
Societies, but the future has yet to determine the
precedence of fundamental additions, such as (a)
consultant and specialist services, (b) institutional
treatment, or (c) the inclusion of the dependants of
insured persons. The policeman's lot may not be a
happy one, but it may be beatific as compared with
that of the Chancellor of the Exchequer who is called
upon to finance any or all of such developments.
But an enactment of the gravest importance to
us as members of the medical profession, whether
general practitioners or consultants, has arrived from
another angle. Whilst our eyes may have been fixed
in watchful regard on the movements of the Health
Insurance Scheme, the Local Government Act, 1930,
camouflaged in the wrappings of a de-rating scheme,
has been placed on the statute book by the late
Government. Had it been introduced by a Labour
Government or ? happy dream ! ? by a Liberal
Government, we might have been more concerned;
but there it is, and you in Bristol, in common with
other centres, have been discussing methods of
adjustment to a measure which in its scope and in
its effect on all branches of the profession is likely
92 Sir Ewen J. Maclean
to prove a more profound revolution than even the
Insurance Acts.
The sections of the Act which concern us principally
are those which impose on the local authorities the
duty of making institutional provision for the sick
within their boundaries ; and under Section 13 in
particular, before deciding as to the kind and amount
of accommodation required to meet their obligations
under the Act, they must consult duly accredited
representatives of the voluntary hospitals in their
areas. An important consideration is that what has
been termed the "taint" of being treated in Poor Law
hospitals has been removed, and irrespective of class
or category the doors of the hospitals under municipal
control are open to all who need the treatment which
these hospitals can provide?subject to the recovery
of actual per capita cost?if necessary by legal process
?from those whose financial position can so afford.
This proviso at once raises the question of the economic
status of the patients in our voluntary hospitals and
the consequential difficulties of transfer from a
voluntary to a municipal hospital and vice versa. It is
obvious, of course, that in any valid scheme of
co-operation between the two classes of hospitals
facilities for transfer must be provided.
This difficulty is especially exemplified in the case
of a patient coming into a voluntary hospital under
the unwritten but admitted obligations of one of the
group contributions by workmen, which are now so
important a factor in the income of the voluntary
hospitals. If such a patient be, for any reason,
transferred to a municipal hospital (other than an
infectious diseases hospital) he incurs the cost of his
Some Reminiscences and a Forecast 93
maintenance therein subject to his proved ability to
pay?whereas whilst he was in the voluntary hospital
he is exempt from payment of any kind or from any
inquiry as to his means. It is clear that difficulty
with the group contributors will arise, not to mention
that of obtaining the consent of the patient to the
transfer. The per contra case of transfer from municipal
hospital to voluntary hospital of a patient for whom
the local authority accepts responsibility will involve
payment of consolidated charges, including the cost
of maintenance plus a sum which would go to a medical
staff fund in accordance with a principle now generally
approved. Personally, I do not think that much help
can be expected from the establishment of district
hospital benefit insurance societies such as have been
proposed. To be adequate the premiums would have
to be substantial, and neither the local authorities nor
the voluntary hospitals would be willing to bear the
insurance risk.
For some such reasons as the foregoing, as well as
other considerations, it is likely that for some time to
come the degree of direct co-operation between the
State and the voluntary hospitals will not be great.
The grade of treatment in comparable institutions
and what is called " the atmosphere " of voluntary
hospitals will continue to maintain their popularity,
but a very important further attraction to the patients
is the low cost?the bulk of them pay little or nothing
?and where a large hospital receives a few pence
or a few shillings per week from other than ticket
or group-contribution patients it does not amount to
much. Just how long is this minimal cost to the
patients in the typical voluntary hospitals to continue ?
94 Sir Ewen J. Maclean
Some will answer, just so long as the legacies, annual
subscriptions, special donations and group contribu-
tions collectively remain sufficient for the purpose.
This is true, but not the whole truth. Incidentally, I
think that the group contributions will increase, whilst
legacies and individual annual contributions will tend
to decrease. But in my judgment a more important
determining factor is the unpaid services of the
honorary staff. I am very far from even suggesting
that the question of the substitution of a salaried
service should now be raised. Nothing could be more
inopportune. But if and when, owing to the
utilization of municipal hospitals, the services of the
honorary staff of the voluntary hospitals cease to have
their counterpart in private practice a new position
will have arisen. Personally I think that epoch is
far from imminent, and that those hospitals will be,
in any re-arrangement, the more favourably placed
which are connected with medical schools.
I am certain that the importance of a large
voluntary hospital as a financial mainstay in a
commercial centre is not sufficiently realized. Taking
the annual value of the worker to the community as
so much, the extent to which he and his comrades are
treated in such a hospital and returned to work
represents to the community a sum equal to the whole
income of the hospital manifolded. In other words,
the community as a whole invests in the hospital as a
going concern a sum equal to the hospital income and
receives back yearly in the shape of restored workers
the equivalent in money value of a large multiple of
that income. An astonishing statement?but capable
of actuarial confirmation. Imagine, then, the financial
Some Reminiscences and a Forecast 95
chaos of the city of Bristol were its two great hospitals
to close their doors.
In this connection it is readily computed that on
a moderate estimate the market value of services
rendered by the honorary staff of a general hospital
of, say, 350 beds is about ?350,000 to ?400,000 per
annum. I am not submitting that in the alternative
that sum divided by the number of the members of
the honorary staff would represent their annual
incomes. But I do say that herein is the great factor
of invisible income upon which, in a given community,
the outstanding financial status of the voluntary
hospital is based. So long as this remains constant
it will be to the economic advantage of employers and
workers to continue and, as occasion requires, to
increase their contributions. Local authorities, too,
will I believe continue, under prevailing conditions,
to make those block grants which have in later years
become almost general, rather than embark upon
schemes involving costly outlay in structure and
salaried service. On this latter point, however,
much will depend, in different areas, upon the
extent to which commitments have already been
incurred.
A financial consideration of some importance to
our voluntary hospitals is that in other countries,
notably in Canada and the United States, much more
than in the British Isles, the addition of a wing for
private patients has proved to be a sound and even a
lucrative investment for the hospital funds, and there
is a growing body of opinion that such a development
in our midst is overdue. Well-conducted and well-
equipped nursing homes and private hospitals would
96 Some Reminiscences and a Forecast
withstand the competition. Others?and there are
too many of such?would not.
I am convinced that there is a possibility that the
municipal authorities in certain districts may be first
in the field.
In my belief, recent legislation notwithstanding,
the days of our voluntary hospitals in their priceless
services to the community, including medical education
and research, are not numbered. Rather is it essential
that they shall continue to flourish, and to preserve
that sanity of outlook and enterprise which, as some
of us know from personal observation in other lands,
has endeared and ennobled them in the view of our
colleagues the world over.
Some of the points I have dealt with?though all
too scantily?Mr. President, are unquestionably of
deep interest to all of us. So much so that in order
to give myself the advantage of knowing the views of
members present I am tempted to beg you to depart
from precedent and to allow some general discussion
to follow.
Once again, sir, may I express my warm thanks
for the honour of being invited to address the Society..

				

## Figures and Tables

**Fig. 1. f1:**
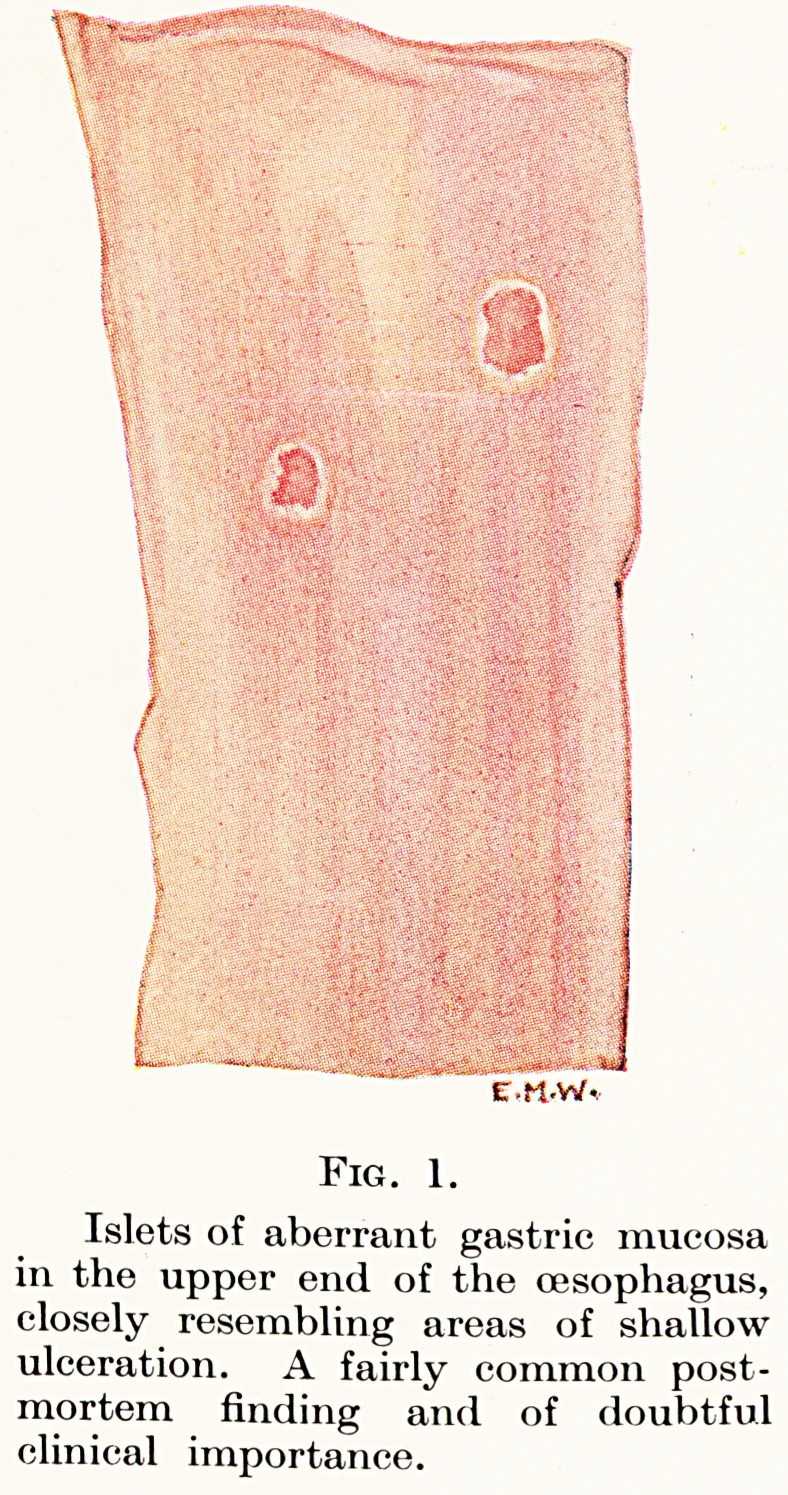


**Fig. 4. f2:**
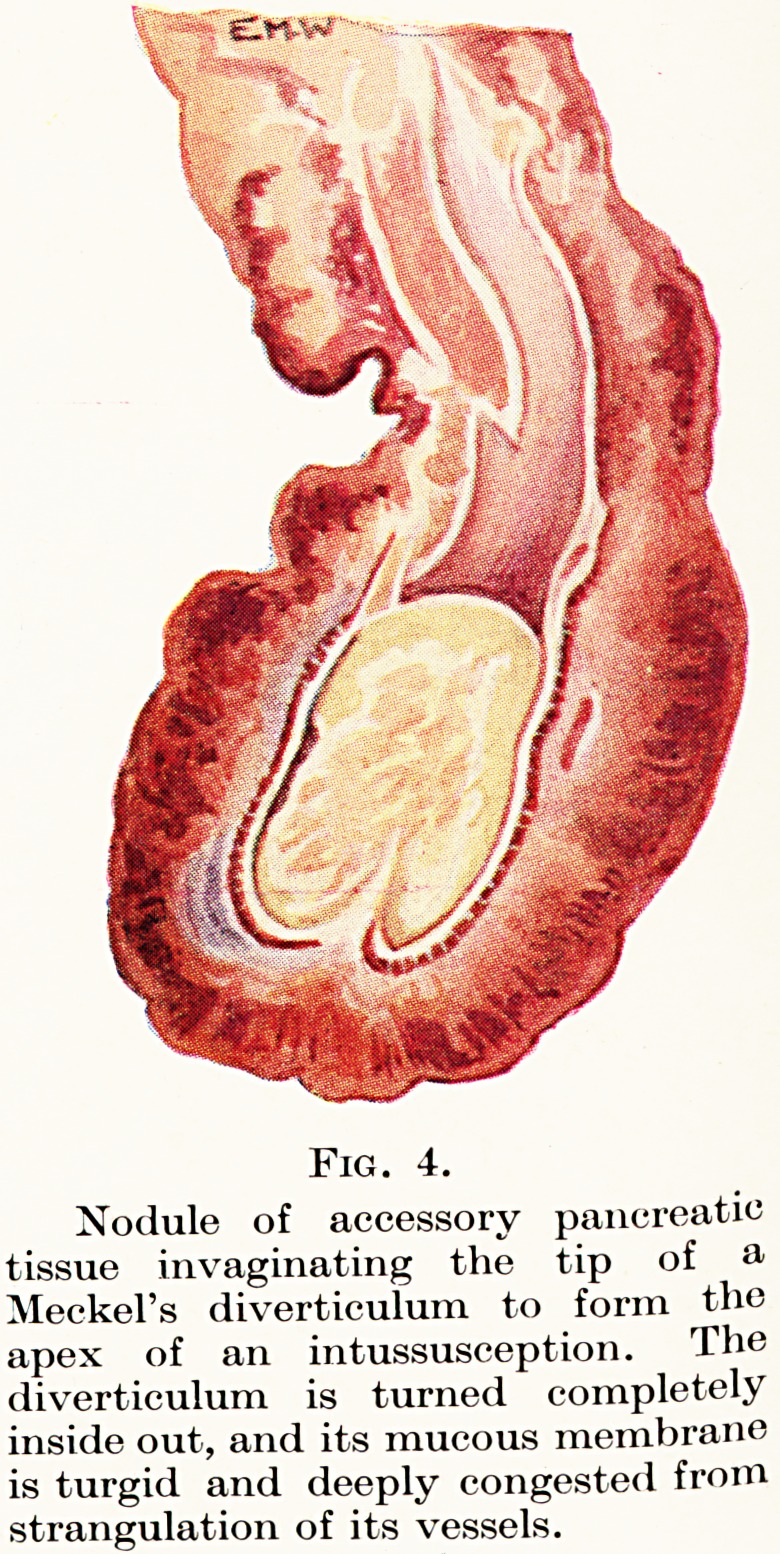


**Figs. 5 and 6. f3:**
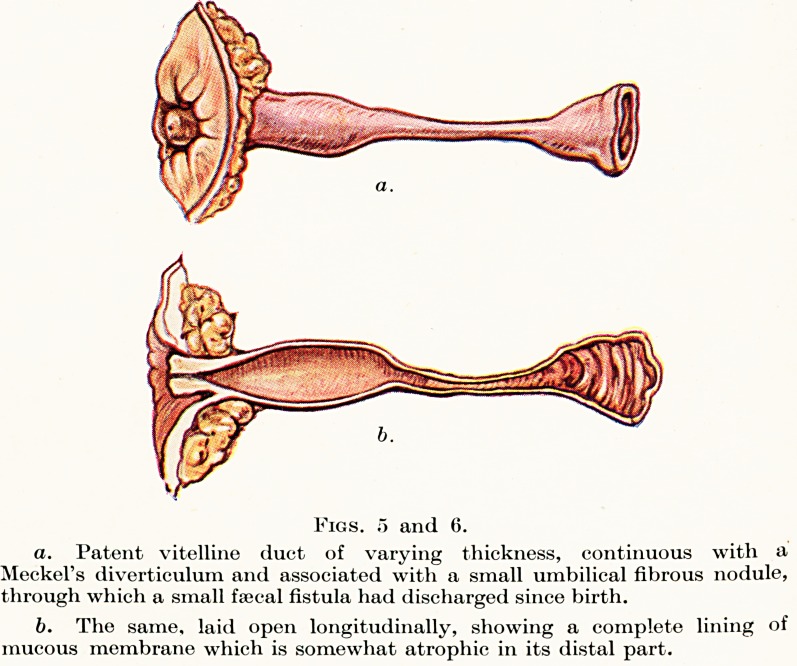


**Fig. 2. f4:**
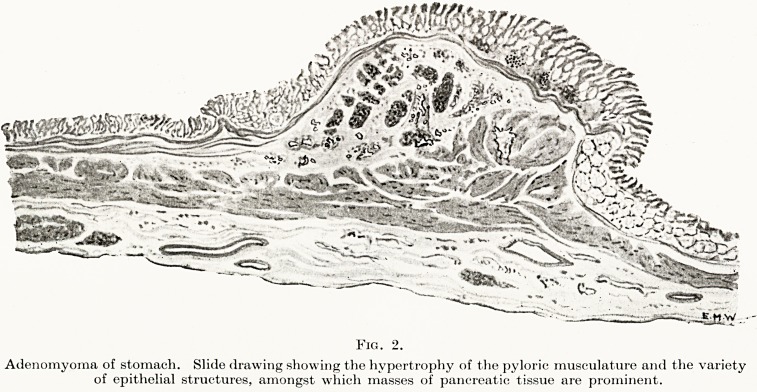


**Fig. 3. f5:**
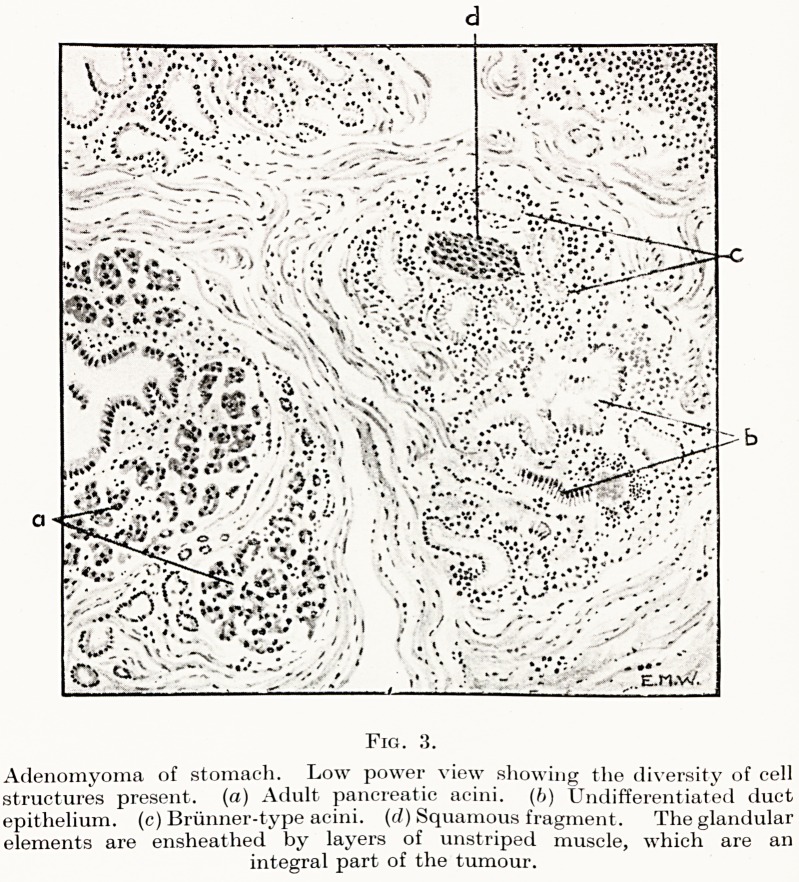


**Fig. 7. f6:**